# Cyclin-Dependent Kinase Inhibitors KRP1 and KRP2 Are Involved in Grain Filling and Seed Germination in Rice (*Oryza sativa* L.)

**DOI:** 10.3390/ijms21010245

**Published:** 2019-12-30

**Authors:** Abolore Adijat Ajadi, Xiaohong Tong, Huimei Wang, Juan Zhao, Liqun Tang, Zhiyong Li, Xixi Liu, Yazhou Shu, Shufan Li, Shuang Wang, Wanning Liu, Sani Muhammad Tajo, Jian Zhang, Yifeng Wang

**Affiliations:** 1 State Key Lab of Rice Biology, China National Rice Research Institute, Hangzhou 311400, China; threetriplea@yahoo.com (A.A.A.); tongxiaohong@caas.cn (X.T.); wangyingkai2006@126.com (H.W.); zhaojuan521321@163.com (J.Z.); liquntang2013@126.com (L.T.); lzhy1418@163.com (Z.L.); 18338690086@163.com (X.L.); mm123456m@126.com (Y.S.); 13126890093@163.com (S.L.); a778211546@163.com (S.W.); dearliuwanning@126.com (W.L.); sanimaimota@gmail.com (S.M.T.); 2 Biotechnology Unit, National Cereals Research Institute, Badeggi, Bida 912101, Nigeria; 3 College of Life Science, Yangtze University, Jingzhou 434025, China

**Keywords:** rice (*Oryza sativa* L.), cyclin-dependent kinase inhibitors, grain filling, seed germination

## Abstract

Cyclin-dependent kinase inhibitors known as KRPs (kip-related proteins) control the progression of plant cell cycles and modulate various plant developmental processes. However, the function of KRPs in rice remains largely unknown. In this study, two rice KRPs members, *KRP1* and *KRP2*, were found to be predominantly expressed in developing seeds and were significantly induced by exogenous abscisic acid (ABA) and Brassinosteroid (BR) applications. Sub-cellular localization experiments showed that KRP1 was mainly localized in the nucleus of rice protoplasts. *KRP1* overexpression transgenic lines (*OxKRP1*), *krp2* single mutant *(crkrp2)*, and *krp1/krp2* double mutant *(crkrp1/krp2)* all exhibited significantly smaller seed width, seed length, and reduced grain weight, with impaired seed germination and retarded early seedling growth, suggesting that disturbing the normal steady state of KRP1 or KRP2 blocks seed development partly through inhibiting cell proliferation and enlargement during grain filling and seed germination. Furthermore, two cyclin-dependent protein kinases, CDKC;2 and CDKF;3, could interact with KRP1 in a yeast-two-hybrid system, indicating that KRP1 might regulate the mitosis cell cycle and endoreduplication through the two targets. In a word, this study shed novel insights into the regulatory roles of KRPs in rice seed maturation and germination.

## 1. Introduction

In eukaryotes, the cell cycle is strictly regulated by cyclin-dependent kinases (CDKs), a type of Ser-Thr protein kinases, together with specific cyclin (CYC) partners during plant growth and development [1,2]. As the central regulators of cell proliferation, the activity of CDKs is finely modulated by numerous molecular mechanisms, including post-translational modification (PTM) such as phosphorylation, proteolysis, and other regulatory proteins [3,4,5,6]. Among these regulatory proteins, CDK inhibitors (CKIs) directly bind to CDK/CYC complexes and inhibit their activities, thereby arresting the cell cycle in response to internal and external cues [7,8,9].

CKIs in mammals are classified into INK4(CKIs, such as p15^INK4b^ and p16^INK4a^ that bind the CDK4 and CDK6 proteins and reduce their binding affinity for cyclin D) and Kip/Cip (CKIs including p21 (Cip1) and p27 (Kip1), that inhibits CDK activity by interaction with both subunits of the CDK–CYC complex) families based on their structural and functional differences [8,10]. The INK4 proteins, containing four or five ankyrin repeats, exclusively bind G1(Gap 1) phase-specific CDKs with D-type cyclins such as CDK4 and CDK6. The Kip/Cip inhibitors, binding to both CDKs and cyclin through a conserved N-terminal domain, show a broader spectrum of inhibitory effects on CDKs with E or A-type cyclins, especially G1 and S (Synthesis) phase CDK/CYC complexes. The plant CDK inhibitors, harboring a conserved 40 amino acids CDK/CYC-binding domain located at the C-terminal region, are similar to the mammalian Kip/Cip family and are therefore designated as Kip-related proteins (KRPs) [11,12]. In *Arabidopsis*, seven KRP family members (*Arath*; KRP1–7) have been discovered through a yeast two-hybrid system, and they block the cell cycle by exclusively inhibiting CDKA and CYCD during the G1/S and G2(Gap 2/M(Mitotic) phases [9]. The overexpression of *Arath;KRP1* showed dwarf phenotype inhibited leaf growth with a decreasing cell number and an up-regulating of the cell size, suggesting that *Arath;KRP1* plays a key role in determining plant growth and development through regulating cell proliferation [13]. The functions of KRPs have also been characterized in tobacco and tomato in the past decade. NtKIS1a, a KRP family member identified in tobacco (*Nicotiana tabacum*), interacts with A-type CDK and D-type cyclins and inhibits the activity of the CDK/cyclin complexes. The overexpression of *NtKIS1a* in *Arabidopsis* inhibits cell division and blocks endoreduplication in nuclei with severe developmental abnormalities, including smaller/shorter vegetative and reproductive organs, strong leaf serration, and enlarged cells [14]. *LeKRP1*, another KRP inhibitor isolated in tomato (*Lycopersicon esculentum*), mainly accumulates in the jelly-like locular (gel) tissue and inhibits CDK/CYC kinase activities in endoreduplicating cells, thereby arresting the mitotic activities to determine the gel tissue development [15].

KRPs regulating cell proliferation/enlargement and plant morphogenesis have also been reported in rice [16,17,18]. Previous studies have identified seven KRP family members in *Japonica* rice (*O. sativa subspecies japonica*), which are designated as *Orysa*;KRP1 to *Orysa*;KRP7, respectively [16,17,19]. Among these KRP inhibitors, *Orysa*;KRP1 to *Orysa*;KRP6 contain the C-terminal CDK/CYC-binding region, which is missing in *Orysa*;KRP7, suggesting that *Orysa*;KRP1 to *Orysa*;KRP6 act as functional CDK inhibitors in rice [17,19]. The overexpression of *Orysa;KRP1* down-regulates grain filling through inhibiting endosperm cell endoreduplications [16]. *Orysa;KRP3* is highly expressed in the cellularized endosperm at two days after fertilization (DAF), while it is not detected in the caryopsis at 3 DAF, suggesting that *Orysa*;KRP3 regulates the cell cycle of syncytial endosperm development [18]. Interestingly, OsiICK1 (corresponding to *Orysa*;KRP1) and OsiICK6 (corresponding to *Orysa*;KRP4) are also reported as functional CDK inhibitors in *Indica* rice (*O. sativa subspecies indica*), and subcellular localization analysis shows that OsiICK1 and OsiICK6 are both mainly localized in the nucleus of transiently transfected tobacco cells, and they could interact with OsCYCD and CDKA through yeast two-hybrid assay [19]. The overexpression of *OsiICK6* significantly reduces plant growth, pollen viability, and seed setting rate. The leaves of *OsiICK6* overexpressing lines roll toward the abaxial side, suggesting that the maintenance of an even growth along the dorsal-ventral plane of leaf blades is at least partially regulated by OsiICK6-mediated cell proliferation [19]. Despite these observations, our understanding of monocot CDK inhibitors is still poor, especially in rice. Here, we characterized two rice cyclin-dependent kinase inhibitors, KRP1 and KRP2, to investigate their tissue-specific expression patterns, the function of the transgenic plants in seed development, and the target CDKs specifically regulated by KRP1, which would advance our understanding of the role of KRPs in rice seed maturation and germination.

## 2. Results

### 2.1. Tissue-Specific Expression Pattern of KRP1 and KRP2

**Figure 1:**
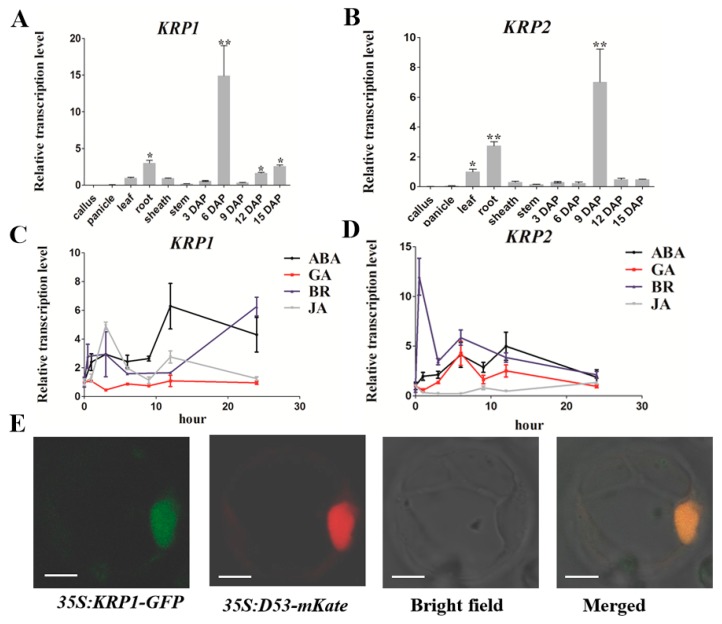
Expression pattern of *KRP1* and *KRP2*. (**A**,**B**) qRT-PCR analysis of *KRP1* and *KRP2* transcription abundances in various rice tissues, respectively. Asterisks indicate the significant difference of other tissues compared with callus, as determined by Student’s *t*-test analysis: * *p* < 0.05. ** *p* < 0.01; (**C**,**D**) qRT-PCR analysis of *KRP1* and *KRP2* transcription abundances in response to various phytohormone treatments, respectively. ABA: abscisic acid; GA: gibberellic acid; BR: brassinosteroids; JA: Jasmonic acid; (**E**) subcellular localization of KRP1–GFP fusion protein in rice protoplasts. D53 fused with mKate was used as a nuclear marker as described [20]. Bar = 10 µM. Error bars indicated SD with biological triplicates (*n* = 3) in (**A**–**D**). Primers used for qRT-PCR analysis were listed in Table S1.

To explore the tissue expression pattern of *KRP1* (*LOC_Os02g52480*) and *KRP2* (*LOC_Os06g11050*), quantitative RT-PCR (qRT-PCR) was performed to examine the transcriptional level of the two CDK inhibitors in eleven different rice tissues. As shown in Figure 1, *KRP1* was predominantly expressed in roots and developing seeds (6, 12, 15 DAP, Day after Pollination), especially in 6 DAP, while extremely low in callus, panicle, and stem (Figure 1A).

*KRP2* exhibited a similar tendency as *KRP1* with higher mRNA accumulation in leaf, root, and 9 DAP during seed development (Figure 1B). However, the transcription levels of *KRP1* and *KRP2* in response to various exogenous phytohormones treatments (100 μM ABA, 100 μM GA, 10 μM BR, and 100 μM JA) at 0, 1, 3, 6, 9, 12, and 24 h were also examined (Figure 1C,D). Upon ABA treatment, the transcription level of *KRP1* increased from one to three hours, then dropped at six hours and kept steady until nine hours, and sharply rose to about six and four-fold at 12 and 24 h, respectively (Figure 1C). In response to BR treatment, the transcription level of *KRP1* increased to two-fold at three hours, then decreased back to a low level at 6 and 12 h and increased to six-fold at 24 h (Figure 1C). For the treatment of JA, *KRP1* increased sharply to the highest point at three hours about five-times-fold, then gradually decreased to two-fold at six hours, and dropped to the basal level at 24 h (Figure 1C). However, *KRP1* showed no response to GA treatment (Figure 1C). As for *KRP2*, all the applied phytohormones, except JA, significantly induced the transcription level of *KRP2*, especially in early stages (Figure 1D). For example, *KRP2* was obviously induced by BR treatment to 15-fold at one hour, 4.1-fold at six hours in response to ABA treatment and 4.3 fold at six hours under GA treatment (Figure 1D). Notably, the high responses of *KRP1* and *KRP2* in transcription strongly indicated that the two KRPs are functionally relevant to ABA and BR. In addition, the subcellular location of KRP1 is likely in the nucleus of rice protoplast, as it co-localized with the nucleus marker D53 [20] (Figure 1E and Figure S1). Given that the plant cell cycle is widespread in the whole-life-cycle, the temporal and spatial expression pattern of *KRP1* and *KRP2* suggests that the two KPR members play important roles in the plant cell cycle, specifically in organ development and hormone-mediated morphogenesis processes.

### 2.2. Functional Characterization of KRP1 and KRP2 in Rice Grain Filling

**Figure 2:**
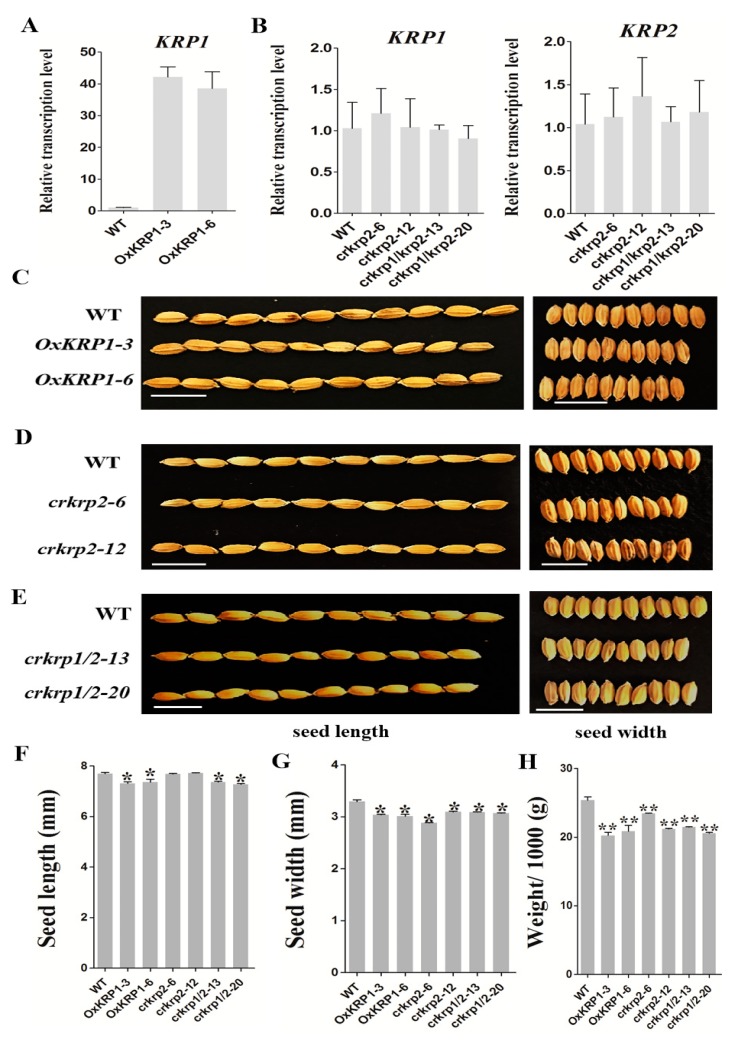
Phenotypic characteristics of *OxKRP1*, *crkrp2*, and *crkrp1/2* mature seeds. (**A**) qRT-PCR analysis for transcript accumulation of *KRP1* in the 6 DAP developing seeds of *OxKRP1* transgenic lines. Error bars indicate the SD with biological triplicates (*n* = 3); (**B**) qRT-PCR analysis for transcript accumulation of *KRP1* and *KRP2* in the 6 DAP developing seeds of *crkrp2* and *crkrp1/2* mutants. Error bars indicate the SD with biological triplicates (*n* = 3); (**C**) comparison of mature seeds of the WT and *OxKRP1* plants. *OxKRP1-3* and *OxKRP1-6* represent two independent *KRP1*-overexpressing lines, respectively; and (**D**) comparison of mature seeds of the WT and *crkrp2* plants. *crkrp2-6* and *crkrp2-12* represent two independent *crkrp2* mutants, respectively; (**E**) comparison of mature seeds of the WT and *crkrp1/2* plants. *crkrp1/2-13* and *crkrp1/2-20* represent two independent *crkrp1/2* double mutants, respectively; (**F**–**H**) seed length, seed width and 1000-seed weight of the WT, *OxKRP1*, *crkrp2*, and *crkrp1/2* mature seeds. Error bars indicate the SD with 50 biological replicates (*n* = 50). Asterisks indicate the significant difference between the WT and transgenic lines, as determined by Student’s *t*-test analysis: * *p* < 0.05, ** *p* < 0.01. WT: wild type, Bar = 1 cm in (**C**–**E**).

The higher expression level of *KRP1* and *KRP2* in developing seeds intrigued us to speculate that the KRP1 and KRP2 might function in the grain filling process (Figure 1A,B). To verify this hypothesis, we generated *KRP1* independent overexpression transgenic lines (*OxKRP1*) with a substantially increased transcription level, *krp2* single mutant (*crkrp2*), as well as *krp1/krp2* double mutant (*crkrp1/krp2*) by using the CRISPR/Cas9 technique (Figure 2A and Figure S2) [21].

Unfortunately, we did not obtain the *krp1* single mutant due to the defects in the CRISPR/Cas9 design. *krp2* single mutants were identified with a T insertion at the first exon of *KRP2* in *crkrp2-6*, an A insertion at the first exon of *KRP2* in *crkrp2-12*, and *crkrp1/krp2* showed a T deletion at the first exon of *KRP1* and a C insertion at the first exon of *KRP2* in *crkrp1/krp2-13*, and three bases GTT deletion at the first exon of *KRP1* and a T insertion at the first exon of *KRP2* in *crkrp1/krp2-20*, resulting in premature termination by shifting the open reading frame (Figure S2). Though the transcriptional level of the mutated genes remained at the same level as native *KRP1* or *KRP2* in the wild type, the insertion/deletion or premature mutation should have disrupted the function of the resulting proteins (Figure 2B).

Under normal conditions, both the grain size of *OxKRP1-3* and *OxKRP1-6* lines were significantly reduced compared with that of the wild-type. The grain length of the wild type was about 7.7 mm, whereas the grain length of *OxKRP1-3* and *OxKRP1-6* decreased to 7.32 mm and 7.37 mm, respectively. Meanwhile, the seed width of *OxKRP1-3* and *OxKRP1-6* dropped to approximately 95% of the wild-type (Figure 2C,F,G). However, the grain size of *crkrp2* and *crkrp1/krp2* also displayed a similar tendency as *KRP1* overexpression lines, though the seed length of *crkrp2* exhibited no significant difference compared to that of the wild-type (Figure 2D–G). Consequently, the 1000-grain-weight of *OxKRP1-3* and *OxKRP1-6* were only 79.0%–82.0% that of the wild-type, and the 1000-grain-weight of the *crkrp2* and *crkrp1/krp2* also had a 12% and 18% reduction compared with the wild-type due to the smaller grain size (Figure 2H). Together, these results demonstrated that disturbing the normal steady state of KRP1 or KRP2 blocks grain filling and both the KRP inhibitors play important roles in seed cell proliferation and enlargement.

### 2.3. Functional Characterization of KRP1 and KRP2 in Seed Germination

**Figure 3:**
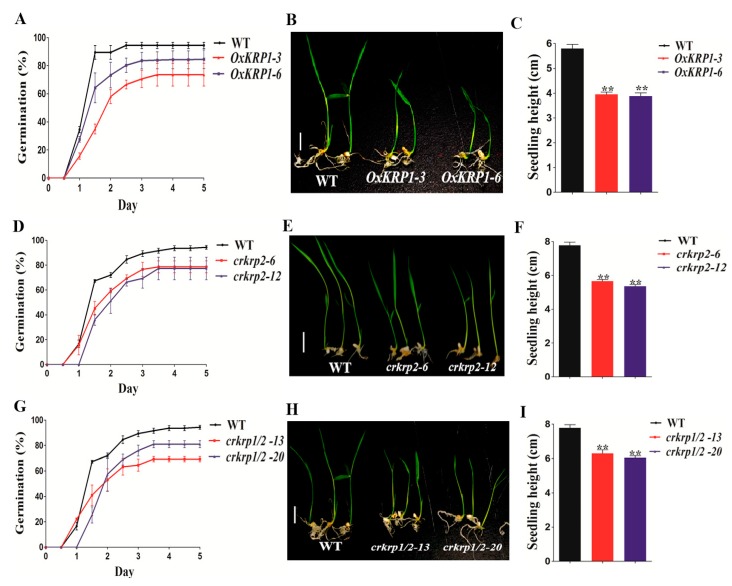
Seed germination characteristics of *OxKRP1*, *crkrp2*, and *crkrp1/2* mutants. (**A**) Germination time courses of the WT and *OxKRP1* on half-strength MS medium, respectively; (**B**) germination phenotypes of the WT and *OxKRP1* growth on half-strength MS medium for 5 days, respectively; (**C**) seedling heights of the WT and *OxKRP1* growth on half-strength MS medium for 5 days, respectively; (**D**) germination time courses of the WT and *crkrp2* mutants on half-strength MS medium, respectively; (**E**) germination phenotypes of the WT and *crkrp2* mutants growth on half-strength MS medium for 5 days, respectively; (**F**) seedling heights of the WT and *crkrp2* mutants growth on half-strength MS medium for 5 days, respectively; (**G**) germination time courses of the WT and *crkrp1/2* mutants on half-strength MS medium, respectively; (**H**) germination phenotypes of the WT and *crkrp1/2* mutants growth on half-strength MS medium for 5 days, respectively; (**I**) seedling heights of the WT and *crkrp1/2* mutants growth on half-strength MS medium for 5 days, respectively. Photographs were taken on day 5, Bar = 1 cm in (**B**,**E**,**H**). Error bars indicate SD with triple biological replicates (*n* = 3, each replicates containing 50 seeds) in (**A**,**D**,**G**). Error bars indicate SD with 50 biological replicates (*n* = 50) in (**C**,**F**,**I**). Asterisks indicate the significant difference between the WT and transgenic lines, as determined by Student’s *t*-test analysis: ** *p* < 0.01 in (**C**,**F**,**I**). WT: wild type, MS: Murashige and Skoog medium.

Since interrupting the equilibrium of KRP1 or KRP2 affects seed maturation, we further investigated their potential roles in seed germination. The seeds of *OxKRP1*, *crkrp2*, and *crkrp1/krp2* mutants all exhibited a slower germination rate when grown on half-strength Murashige and Skoog medium (Figure 3). As shown in Figure 3A, 94.5% of the wild-type seeds germinated in 60 h, whereas 66.7% and 80.3% of the seeds of *OxKRP1-3* and *OxKRP1-6* germinated at the same time point, respectively (Figure 3A).

As for *crkrp2* and *crkrp1/krp2* mutants, the germination rates were 67.8% and 65.6% in 60 h, respectively (Figure 3D,G). Consistent with the seed germination tendency, the post-germination growth of *OxKRP1*, *crkrp2*, and *crkrp1/krp2* were also significantly retarded compared with that of the wild type (Figure 3B,C,E,F,H,I). Additionally, *OxKRP1*, *crkrp2*, and *crkrp1/crkrp2* mutants also displayed retarded early seedlings growth (Figure S3). Taken together, these results strongly suggested that *KRP1* and *KRP2* participate in normal seed germination and early seedling growth.

### 2.4. Grain Filling and Seed Germination Related Genes Regulated by KPR1

**Figure 4:**
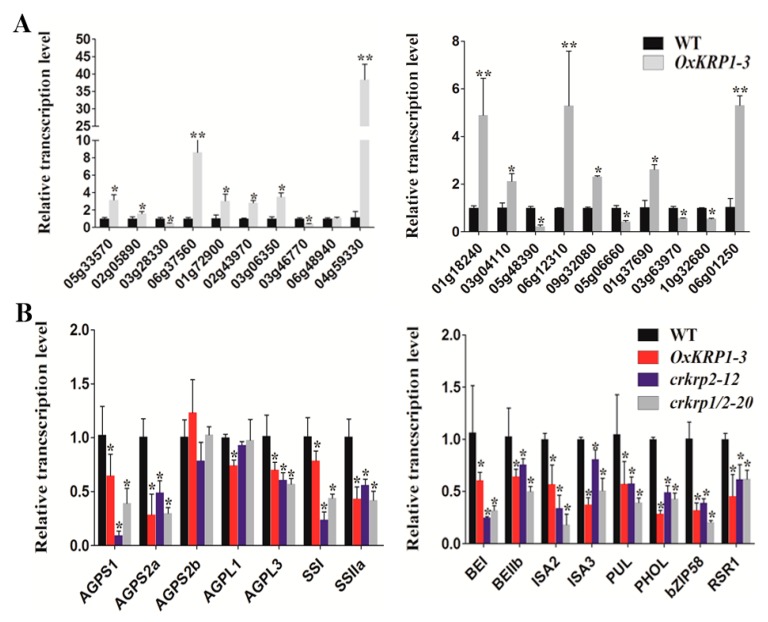
Expression analysis of KPR1 regulated genes by qRT-PCR. (**A**) qRT-PCR validation of the DEGs revealed by RNA-seq experiments; (**B**) qRT-PCR analysis of the transcriptional abundances of grain filling related genes in the WT, *OxKRP1*, *crkrp2*, and *crkrp1/2* mutants. 6 DAP seed cDNA were used as templates for this analysis. DEGs: Differentially Expressed Genes. Error bars indicate SD with biological triplicates (*n* = 3). Asterisks indicate the significant difference between the WT and transgenic lines, as determined by Student’s *t*-test analysis: * *p* < 0.05, ** *p* < 0.01. Primers used in (**A**) were listed in Table S1. Primers used in (**B**) were used according to previous reports [22,23].

To figure out the genes regulated by KRP1, RNA-seq on the wild type and *OxKRP1* developing seeds at 6 DAP (days after pollination) were carried out. As a result, a total of 1710 genes were found to be differentially expressed in *OxKRP1*, including 943 up-regulated genes and 767 down-regulated genes directly/indirectly mediated by KRP1 (|log 2 Ratio| ≥ 1; *p* value < 0.01) (Table S2). To validate the RNA-seq data, 20 DEGs (Differentially Expressed Genes) were selected to analyze the gene transcript abundance (Table S3). As shown in Figure 4A, most of the transcription levels of the chosen genes were consistent with the RNA-seq results, suggesting a high reliability of the transcriptomic data (Figure 4A).

Among these detected DEGs, several have been reported to be functionally involved in rice seed development. For example, *ARAG1* (*AP2/EREBP-type transcription factor*) (*LOC_Os02g43970*), mainly expressed in germinating seeds and strongly induced by drought stress and ABA treatment [24], were up-regulated in *OxKRP1* and exhibited co-expression patterns with *KRP1* [24], indicating that ARAG1 could be involved in KRP1-mediated seed germination. *OsPPDKB* (White-core floury endosperm-4) (*LOC_Os05g33570*), regulating the carbon metabolism during grain filling [25,26], was also induced in *OxKRP1*, suggesting that KRP1 could partly modulate seed development through adjusting carbon distribution, such as starch and fatty acids. Given that some genes with minor, but statistically significant, differences may be undetected by RNA-seq, we further examined the transcription level of numerous key grain filling regulators in *OxKRP1* 6 DAP developing seeds, including ADP-glucose pyrophosphorylase (AGPase), soluble starch synthase (SS), starch branching enzyme (SBE), starch debranching enzyme (DBE), and starch phosphorylase L (PHOL) directly involved in amylose and amylopectin biosynthesis, and two transcription factors regulating rice starch biosynthesis such as *RSR1* (Rice Starch Regulator1, a rice AP2/EREBP family transcription factor) and *bZIP58* (*RISBZ1*, a basic leucine zipper transcription factor) during seed development [23,27]. As expected, most of these key regulators were down-regulated in *OxKRP1*, except for AGPS2b (Figure 4B). Interestingly, we also found the mRNA abundance of these regulators in *crkrp2* and *crkrp1/krp2*, which exhibited a similar tendency to that of *OxKRP1* (Figure 4B). These results well explained the significantly reduced seed size and grain weights in these KRPs related transgenic plants.

To functionally categorize these DEG, GO (gene ontology), and KEGG (Kyoto Encyclopedia of Genes and Genomes) pathway analyses were utilized to obtain an overview of the dynamic transcriptomic change in *OxKRP1*. GO analysis showed that the DEGs were preferentially catalogued into “cell part”, “organelle”, and “membrane”, whereas “symplast”, “cell junction”, and “extracellular region” were under-represented in terms of “cellular component” (Figure S4A and Table S4). From the perspective of “biological process”, these DEGs were majorly related to “cellular process”, “metabolic process”, and “regulation of biological process”, suggesting that metabolism and signal processes are required for KRP1 in plant cell cycle regulation. On the contrary, “pigmentation” and “rhythmic process” only took less than 1% (Figure S4A and Table S4). With respect to “molecular function”, we found that “catalytic activity” and “binding” were over-represented, indicating that numerous signal transduction factors or enzymes are involved in KRP1-mediated seed morphogenesis. However, only less than 1% of the DEGs were related to “transcription regulator activity” and “translation regulator activity” (Figure S4A and Table S4). However, KEGG analysis showed that DEGs related to metabolic pathways, starch, and sucrose metabolism, and the biosynthesis of secondary metabolites were highlighted (Figure S4B and Table S5). Since these pathways were tightly associated with storage compound synthesis and cell proliferation [28], the KEGG results strongly supported that KRP1 may affect seed morphogenesis and nutrient accumulation during grain filling. Additionally, CELLO (http://cello.life.nctu.edu.tw/) [29] was performed to predict the subcellular location of these DEGs. As a result, the DEGs preferentially occurred in the nucleus, plasma membrane, and chloroplast, which represented a proportion of 30.56%, 20.86%, and 15.13%, respectively, while other cellular compartments, such as peroxisomal, ER, and golgi only accounted for 0.46% of these DEGs (Figure S4C). The nuclear subcellular location of a large proportion of these DEGs indicate that KRP1 mainly performs its function in the nucleus, which was in consistent with the specific nuclear location of KRP1 in Figure 1E.

### 2.5. Determination of the Interactions of KRP1 with CDKs

**Figure 5:**
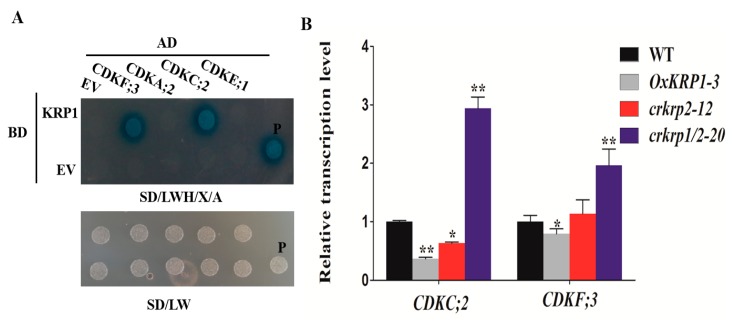
Yeast-two-hybrid assay of KRP1-CDKs interaction. (**A**) Yeast two-hybrid assays. Yeast cells co-transformed with *CDKC;2* or *CDKF;3* fused to the GAL4 activation domain (CDKC;2-AD or CDKF;3-AD) and *KRP1* fused to the GAL4 binding domain (KRP1-BD) were grown on selective media. BD, pGBKT7; AD, pGADT7; EV, empty vector; SD/LW, -Leu-Trp; SD/LWH, -Leu-Trp-His; P, positive control, pGADT7-T+pGBKT7-53; X: X-α-Gal, 0.04 mg/mL; and A: Aureobasidin A, 100 ng/mL. (**B**) qRT-PCR analysis of the transcriptional abundances of *CDKC;2* and *CDKF;3* in the WT, *OxKRP1*, *crkrp2*, and *crkrp1/2* mutants. Error bars indicate the SD with biological triplicates (*n* = 3). Asterisks indicate the significant difference between the WT and transgenic lines, as determined by Student’s *t*-test analysis: * *p* < 0.05, ** *p* < 0.01. WT: wild type. The primers used for qRT-PCR analysis were listed in Table S1.

To search for the KRP1 interactive proteins, four different types of CDKs were selected to investigate their interaction with KRP1 by yeast two-hybrid assay. As shown in Figure 5A, KRP1 was able to interact with CDKC;2 (LOC_Os01g72790) and CDKF;3 (LOC_Os03g63020), but not with CDKA;2 (LOC_Os02g03060) and CDKE;1 (LOC_Os10g42950) (Figure 5A).

Furthermore, the transcription levels of these two CDK members were detected in the 6 DAP developing seeds of *OxKRP1*, *crkrp2*, and *crkrp1/krp2*. As shown in Figure 5B, the transcription level of *CDKC;2* and *CDKF;3* were both significantly down-regulated in *OxKRP1*, while slightly decreased in *crkrp2* (Figure 5B). Interestingly, the transcription levels of *CDKC;2* and *CDKF;3* were drastically increased in *crkrp1/krp2* double mutants (Figure 5B). These results demonstrated that the *CDKC;2* and *CDKF;3* are mainly inhibited by KRP1, while not KRP2 due to the functional difference of the two KRP inhibitors. However, CDKC;2 and CDKF;3 have not been functionally reported until now. Only spatial and temporal expression patterns have shown that *CDKC;2* and *CDKF;3* are expressed in developing seeds (0, 1, 3, 6 DAP, Day After Pollination), endosperms (9, 12, and 15 DAP) and embryos (9 and 15 DAP), as previously reported [17], indicating the potential role of the two CDK members participating in cell cycle regulation during seed development. Future work focusing on how CDKC;2 and CDKF;3 are involved in KRP1-governed grain filling and seed germination would clarify the finely regulated mechanisms of KRP1.

## 3. Discussion

In the past decades, several KRP inhibitors have been reported in connection with the cell cycle and plant morphogenesis. In *Arabidopsis*, ectopically overexpression of *Arath;KRP1* under different organ-specific promoters, such as trichrome-specific *GL*2, petal-specific *AP3*, and pollen-specific *Bgp1* promoters, usually inhibit the relative vegetative and reproductive organs development and lead to abnormal morphogenesis, such as impaired fertility, smaller size and reduced number of branches, and collapsed trichrome [30,31]. In monocotyledonous plants, overexpression of *Orysa;KRP1* plants in *Japonica* rice decreased endoreduplication and thereby reduced grain filling, and overexpression of *OsiICK6* in *Indica* rice also impaired vegetative growth and reduced seed production, similar to the phenotype observed in the overexpression plants of *Arath;KRP1* driven by a constitutive promoter [12,13,14,16,19,32]. Two maize *KRP* inhibitors, *Zeama;KRP1* and *Zeama;KRP2*, are characterized by the inhibiting the activities of CDK and endoreduplication during endosperm development [33]. However, the biological functions of these KRP inhibitors are mainly obtained through the phenotypic analysis of the overexpression transgenic plants, while far less was done on the mutants. In this study, *KRP1* overexpression plants drastically reduced seed production through down-regulating the seed length and width (Figure 2C,F–H). Interestingly, we also discovered that the seed morphological characteristic of *krp2* single (*crkrp2*) and *krp1/krp2* double mutants (*crkrp1/krp2*) were largely the same as that of *KRP1* overexpression plants (Figure 2D–H), suggesting that either overexpression or mutation of *KRPs* could disrupt the normal steady state of the two cell cycle regulators, thereby affecting cell proliferation during seed development. Moreover, most of the reported grain filling related genes were significantly down-regulated in either *OxKRP1* or *crkrp2* and *crkrp1/krp2* developing seeds (Figure 4B), which explained well the reduced seed size and grain weight in these transgenic plants. However, slower seed germination and early retarded seedling growth were detected in *OxKRP1*, *crkrp2*, and *crkrp1/krp2* mutants, possibly due to the seed morphogenesis activity blocked by KRP1 or KRP2 (Figure 3 and Figure S3). Notably, *crkrp2* and *crkrp1/krp2* both drastically reduced grain filling and seed germination, which reduced the functional redundancy between the two KRP members, though KRP1 and KRP2 displayed 51% amino acid sequence similarity and belonged to the same subgroup based on the phylogenetic analysis in a previous report [16].

Subcellular location is important for cell cycle regulators to perform their functions [34,35]. The tobacco CDK inhibitors NtKIS1a and NtKIS2 and all seven *Arabidopsis* KRP inhibitors are localized in the nucleus, and the punctuate pattern of sub-nuclear distribution is determined by the conserved protein motif ‘YLQLRSRRL’, in which the third residue is variable [19,36,37]. In this study, we first validated that KRP1 was specifically co-localized in the nucleus with the nuclear marker D53 [20] in rice protoplasts (Figure 1E), which strongly implied that KPR1 could act as a nuclear protein and interact with the downstream targets to mediate cell proliferation and plant organ development, which is also in consistent with the conserved nuclear localization of plant KRP proteins [19]. Additionally, KRP1 displayed a more homogeneous sub-nuclear punctuate pattern (Figure 1E), a partly conferred by the conserved sequence ‘YLQLRSRML’ located in KRP1, and a similar phenomenon is also detected in the sub-cellular localization of OsiICK1 in the genetic background of *Indica* rice [19].

Accumulating evidence has shown that CDK inhibitors could connect the phytohormones with cell cycle modulation in response to environmental cues. For example, previous studies have shown that *KRP1* is induced by abscisic acid (ABA), while *KRP2* involved in lateral root initiation is down-regulated by auxin in *Arabidopsis* [38,39,40]. *KRP5* is up-regulated by auxin, while three KRP members, *KRP1*, *4*, and *5*, are inhibited by cytokinin [17]. *OsiICK6* is also induced by ABA, which is similar to its *Arabidopsis* homology [38]. In this study, we determined that *KRP1* and *KRP2* were preferentially induced by ABA and BR (Figure 1C,D). Given that ABA and BR extensively regulate seed maturation and germination, as previously described [41,42,43,44,45,46], these results demonstrated that ABA and/or BR could affect the two cell cycle regulators by regulating cell proliferation during grain filling and seed germination processes.

In this study, *KRP1* and *KRP2* were highly expressed in 6 DAP and 9 DAP, respectively (Figure 1A,B). During seed developmental stages, the seed embryo differentiates into apical meristem and leaf primordium, and the endosperm cells gradually turn into aleurone cells and starch storage cells, and terminate cell division at around 9–10 DAP [16,47,48]. The spatial and temporal expression pattern showed that KRP1 and KRP2 were tightly involved in seed rapid cell proliferation and could act as key switches from the mitotic cell cycle to the endocycle. Additionally, previous studies have reported that the overexpression of *KRP1* drastically inhibits endoreduplication by turning the number of nuclei from 12–24 C to 3 C ploidy level during endosperm formation, ultimately down-regulating seed nutrient absorption and yield [16,49]. Since *crkrp2* also exhibits similar seed production as *OxKRP1*, it would be interesting to further figure out whether KRP2 also regulates endosperm development by hindering endoreduplication.

In the past decades, several reports have shown that KRPs directly bind and inhibit cyclin/CDK complexes and impair the latter’s activity in plants [9,14,15,18,19,32,33,38,50]. Based on the phylogenetic analysis of the catalytic site and cyclin-binding motifs, rice CDKs could be divided into eight different types (A–G-type and CKL-type), similar to CDKs in *Arabidopsis* [17,51]. Among these different types of CDKs, A-type CDKs (CDKA) are characterized by the PSTAIRE motif located in the cyclin-binding domain, C-type CDKs (CDKC) harbours the canonical PITAIRE motif, E-type CDKs (CDKE) is defined by its conserved SPTAIRE motif, and F-type CDKs (CDKF) could phosphorylate the threonine residue in the T-loop of other CDKs, as previously reported [2,52,53]. However, the target CDKs regulated by KRP1 in rice is still unclear. In this study, we selected four representatives CDKs to detect whether these CDK members could interact with KRP1. To our surprise, KRP1 directly interacts with CDKC;2 or CDKF;3, but not with CDKA;2 or CDKE;1 through yeast two-hybrid assay (Figure 5A). Previous studies have shown that plant KRP inhibitors mainly inhibit A-type CDK and D-type cyclins complexes [18,19,32,50]; the protein–protein interaction results suggest the functional differences of KRP1 with other KRP inhibitors in terms of the interaction with OsCDKs. Consistent with the yeast two-hybrid results, the transcription level of *CDKC;2* and *CDKF;3* were reduced in *OxKRP1* developing seeds (Figure 5B). Additionally, *CDKC;2* and *CDKF;3* were detected in developing seeds (0, 3, and 6 DAP), as previously reported [17], which were overlapped with the tissue expression pattern of *KRP1* (Figure 1A). These results suggest that KRP1 could down-regulate CDKC;2 or CDKF;3, and future work addressing how KRP1 regulating the activates of CDKC;2 and/or CDKF;3 will advance our understanding of the molecular mechanism of KRP1 involvement in rice grain filling and seed germination.

## 4. Materials and Methods

### 4.1. Subcellular Localization Analysis

To generate the rice protoplast, 5 g of rice leaf strips (grown in dark for two weeks) in 0.5 mm size were digested in 10 mL enzyme solution (1.5% cellulose R10, 0.75% macerozyme R10, 0.6 M mannitol, 10 mM MES pH = 7.5) for 6 h in the dark with gentle shaking (40 rpm) at 28 °C. The protoplasts were filtered and harvested by centrifugation, washed with 10 mL W5 solution (154 mM NaCl, 125 mM CaCl_2_, 2 mM KH_2_PO_4_, 2 mM MES, 5 mM glucose, pH = 5.7) twice, then the protoplasts were suspended in MMG solution (0.4 M mannitol, 15 mM MgCl2, 4 mM MES, pH = 5.8). The full-length the coding region of *KRP1* without the stop codon was amplified and fused with eGFP in p35S-GFP vector and designated as 35S:KRP1-GFP. 35S:KRP1-GFP and 35S:D53-mKate (a nucleus marker) [20] were co-transiently expressed in the rice protoplasts and incubated in PEG (0.6 M mannitol, 100 mM CaCl2, 40% PEG4000) for 30 min at room temperature. The GFP signals were detected by observation using a Zeiss LSM710 laser confocal microscope (Carl Zeiss, AG, Jena, Germany).

### 4.2. Vector Construction and Plant Transformation

The CRISPR/Cas9 system was adopted, as previously described [22]. Annealed double-strand oligos of the gDNA sequences of *KRP1* were ligated into the pYLgRNA-OsU3 using *Bsa*I site (Thermo, Waltham, MA, USA). To make *KPR1* overexpression construct, CDS of *KRP1* were amplified and inserted between the *Kpn*I and *BamH*I sites of vector pU1390, which was driven by a strong constitutive ubiquitin promoter. The primers used were listed in Table S1. All the constructed plasmids were transformed into the rice variety ‘Nipponbare’ (*Oryza sativa* L. cv. Nipponbare) callus using the *Agrobacterium*-mediated transformation method, as previously described [54].

### 4.3. RNA-Seq

The total RNA of 6 DAP rice seeds of the wild type (WT) and *KPR1* overexpression (*OxKRP1*) plants were analyzed by Nanodrop 2000 spectrophotometer (Thermo, Waltham, MA, USA) and Agilent 2100 Bioanalyzer (Agilent, Santa Clara, CA, USA). Three biological replicates were used for each sample. The qualified RNA was processed for sequencing library construction, as previously described [55]. Briefly, the high-throughput sequencing was performed using the Illumina HiSeq™ 2000 platform (Illumina, Foster City, CA, USA) to obtain a standard quality library, reads with low-quality, adaptor-polluted, unknown base (N), and counts below 20 reads per million were deleted. The generated clean reads were aligned with the transcripts of rice genes in RGAP (http://rice.plantbiology.msu.edu/) using BOWTIE 2(http://bowtie-bio.sourceforge.net/bowtie2/index.shtml), and the gene expression level was calculated using RSEM (RNA-seq by expectation maximization) [56]. Differentially expressed genes (DEGs) between the three biological replicates of either the WT and *OxKRP1* were identified using the EBSeq [57], and the cutoff value and fragments per kilobase of transcript per million mapped reads (|log 2 Ratio| ≥ 1; *p* value < 0.01) were used as thresholds to identify significant differences in gene expression.

### 4.4. RNA Isolation and qRT-PCR

The RNA of all the tissues, except developing seeds, was extracted by Trizol (Invitrogen, Carlsbad, CA, USA) according to the manufacturer’s instructions. For developing seeds, a modified SDS-trizol method was applied, as previously described [22]. Briefly, 0.5 g of ground seed powders were treated with 300 µL SDS RNA extraction buffer (50 mM Tris-HCl pH 8.0, 5 mM EDTA pH 8.0, 150 mM LiCl and 1% SDS), then 300 µL Phenol (pH 8.0): Chloroform = 1:1. After centrifugation was added; the RNA in the supernatant was extracted by Trizol according to the manufacturer’s instructions. The total RNA was quantified by nanodrop spectrometer (Thermo, Waltham, MA, USA) and 2 µg of each sample was used for reverse transcription using MMLV reverse transcriptase (Takara, Dalian, China) according to the manufacturer’s instructions. Quantitative RT-PCR (qRT-PCR) was performed with technical triplicates in a total of 10 μL reaction volume containing 5 µL THUNDERBIRD SYBR qPCR Mix (Toyobo, Shanghai, China), 1 µL cDNA, 0.2 µL primers, and 3.8 µL water using CFX96 touch real-time PCR detection system (Bio-rad, Hercules, CA, USA). The relative expression level of tested genes was normalized to the housekeeping gene ubiquitin (GenBank accession No. AF184280) detected in the same sample and was calculated by the 2^−ΔΔCT^ method [58]. The primers used are listed in Table S1.

### 4.5. Yeast-Two-Hybrid Assay

The interactions of KRP1 with different CDKs were analysed using the Matchmaker™ Gold Yeast Two-Hybrid system (Clontech, Dalian, China) according to the manufacturer’s instructions. The CDS of *KRP1* was amplified and cloned into the plasmid pGBKT7 (BD) as the bait and the CDSs of *CDKA;2*, *CDKC;2*, *CDKE;1*, and *CDKF;3* were amplified and cloned into plasmid pGADT7 as the prey. The primers used are listed in Table S1. Different combinations of BD and AD constructs were co-transformed into the yeast strain Y2H Gold (Clontech, Dalian, China). The co-transformed yeast cell was cultured in SD/-Trp-Leu selective medium, and further screened in SD/-Trp-Leu-Ade-His selective medium with 0.04 mg/mL X-α-gal and 30 mM 3-AT to detect the interaction by visualizing the colour of the yeast colonies.

### 4.6. Phenotypic Analysis of Rice Transgenic Plant Seeds

The length, width, and thousand-grain-weight of the WT and all the *KRP*-related transgenic seeds were measured by the seed phenotyping system (Wangsheng, Hangzhou, China) and analyzed by graphpad prism 5 software (Graphpad software, San Diego, CA, USA) according to the manufacturer’s instructions with 50 biological replicates per sample. Seed germination assay was performed, as previously reported [59]. Briefly, the WT, *KRP1* overexpression lines, *krp2* single mutant (*crkrp2*), and *krp1/krp2* double mutant (*crkrp1/krp2*) seeds were first surface sterilized in 70% ethanol for 1 min and soaked in 50% NaClO for 30 min. Then, the seeds were washed in sterilized water for five times and placed on half-strength Murashige and Skoog medium with 0.3% plant agar in a growth chamber (28 ± 2 °C, 12/12 h photoperiod with 60% relative humidity). A growth of 5 mm long coleoptile was considered a complete germination. The seed germination rate was recorded every 12 h for 5 days and triple biological replicates (each replicates containing 50 seeds) were performed for each sample.

In this study, we first systematically reported the biological function of two rice KRP inhibitors, KRP1 and KRP2, involved in grain filling and seed germination. *KRP1* overexpression transgenic lines (*OxKRP1*), *krp2* single mutant *(crkrp2)*, and *krp1/krp2* double mutant *(crkrp1/krp2)* all displayed poor seed production, together with retarded seed germination and impaired early seedling growth, suggesting that both KRP inhibitors play important roles in seed morphogenesis. Consistent with the phenotype of these transgenic lines, a series of grain filling key regulators, such as ADP-glucose pyrophosphorylase (*AGPase*), soluble starch synthase (*SS*), and Rice Starch Regulator1 (*RSR1*), were significantly down-regulated in *OxKRP1*, *crkrp2*, and *crkrp1/krp2.* Furthermore, we first showed that KRP1 was located in the nucleus of rice protoplasts and interacted with two cyclin-dependent protein kinases, CDKC;2 and CDKF;3, through the yeast two-hybrid system, indicating the two CDK members could participate in KRP1-mediated seed development. In a word, our work not only expands the knowledge on the finely regulated mechanism of KRPs, but also provides novel insights into the roles of KRPs in rice seed maturation and germination to facilitate plant functional genomics.

## Supplementary Materials

Supplementary materials can be found at https://www.mdpi.com/1422-0067/21/1/245/s1. Table S1. Sequences of primers used in this study. Table S2. Differentially expressed genes between wild-type and *OxKRP1* transgenic lines. Table S3. Selected DEGs used for RNA-seq verification. Table S4. GO term analysis for Gene Ontology (GO) in this study. Table S5. KEGG pathway analysis in this study. Figure S1. Subcellular localization of KRP1–GFP fusion protein in (**A**) or the nucleus marker D53 in (**B**) in rice protoplasts, respectively. Bar = 10 µM. Figure S2. Molecular characterization of *crkrp2* and *crkrp1/2* mutants. (**A**) Schematic presentation of the gene structure of *KRP1*, *KRP2* and CRISPR-cas9 editing site. PAM: protospacer adjacent motif. Black boxes: untranslated regions; Brown boxes: exons; black line: intron; (**B**) Sanger sequencing chromatograph of the CRISPR-cas9 target site in homozygous mutants of *crkrp2* and *crkrp1/2*, respectively. The letter in red represented the mutant sites. The letter in blue represented the PAM sequence. Figure S3. Early seedlings establishment of *OxKRP1*, *crkrp2* and *crkrp1/2* mutants. (**A**–**C**) Growth phenotype of the WT, *OxKRP1*, *crkrp2* and *crkrp1/2* mutants after cultured in the hydroponic solution as described [60] for two weeks. Bar = 5 cm; (**D**) Seedlings height of *OxKRP1*, *crkrp2* and *crkrp1/2* mutants in correspondence to (**A**–**C**). Error bars indicate SD with ten biological replicates (*n* = 10). Asterisks indicate the significance of differences between the WT and transgenic lines as determined by Student’s *t*-test analysis: ** *p* < 0.01. Figure S4. Functional categorization of the differentially expressed genes (DEGs) between the WT and *OxKRP1* 6 DAP developing seeds. (**A**) Enrichment analysis of the DEGs in Gene Ontology (GO) categories in terms of cellular components, biological processes and molecular functions; (**B**) Enrichment analysis of the DEGs in KEGG pathways; (**C**) Distribution of the DEGs in subcellular compartments.

## References

[1] Morgan D.O. (2007). The Cell Cycle: Principles of Control (Primers in Biology).

[2] Joubes J., Chevalier C., Dudits D., Heberle-Bors E., Inze D., Umeda M., Renaudin J.P. (2000). CDK-related protein kinases in plants. Plant Mol. Biol..

[3] Torres Acosta J.A., Fowke L.C., Wang H. (2011). Analyses of phylogeny, evolution, conserved sequences and genome-wide expression of the ICK/KRP family of plant CDK inhibitors. Ann. Bot. Lond..

[4] Inze D. (2005). Green light for the cell cycle. EMBO J..

[5] Dewitte W., Murray J.A. (2003). The plant cell cycle. Ann. Rev. Plant Biol..

[6] De Veylder L., Joubes J., Inze D. (2003). Plant cell cycle transitions. Curr. Opin. Plant Biol..

[7] Lui H., Wang H., Delong C., Fowke L.C., Crosby W.L., Fobert P.R. (2000). The *Arabidopsis* Cdc2a-interacting protein ICK2 is structurally related to ICK1 and is a potent inhibitor of cyclin-dependent kinase activity in vitro. Plant J..

[8] Sherr C.J., Roberts J.M. (1999). CDK inhibitors: Positive and negative regulators of G1-phase progression. Genes Dev..

[9] Verkest A., Weinl C., Inze D., De Veylder L., Schnittger A. (2005). Switching the cell cycle. Kip-related proteins in plant cell cycle control. Plant Physiol..

[10] Sherr C.J., Roberts J.M. (1995). Inhibitors of mammalian G1 cyclin-dependent kinases. Genes Dev..

[11] Wang H., Fowke L.C., Crosby W.L. (1997). A plant cyclin-dependent kinase inhibitor gene. Nature.

[12] De Veylder L., Beeckman T., Beemster G.T., Krols L., Terras F., Landrieu I., van der Schueren E., Maes S., Naudts M., Inze D. (2001). Functional analysis of cyclin-dependent kinase inhibitors of *Arabidopsis*. Plant Cell.

[13] Wang H., Zhou Y., Gilmer S., Whitwill S., Fowke L.C. (2000). Expression of the plant cyclin-dependent kinase inhibitor ICK1 affects cell division, plant growth and morphology. Plant J..

[14] Jasinski S., Perennes C., Bergounioux C., Glab N. (2002). Comparative molecular and functional analyses of the tobacco cyclin-dependent kinase inhibitor NtKIS1a and its spliced variant NtKIS1b. Plant Physiol..

[15] Bisbis B., Delmas F., Joubes J., Sicard A., Hernould M., Inze D., Mouras A., Chevalier C. (2006). Cyclin-dependent kinase (CDK) inhibitors regulate the CDK-cyclin complex activities in endoreduplicating cells of developing tomato fruit. J. Biol. Chem..

[16] Barroco R.M., Peres A., Droual A.M., De Veylder L., Nguyen L.S.L., De Wolf J., Mironov V., Peerbolte R., Beemster G.T.S., Inze D. (2006). The cyclin-dependent kinase inhibitor orysa; KRP1 plays an important role in seed development of rice. Plant Physiol..

[17] Guo J., Song J., Wang F., Zhang X.S. (2007). Genome-wide identification and expression analysis of rice cell cycle genes. Plant Mol. Biol..

[18] Mizutani M., Naganuma T., Tsutsumi K., Saitoh Y. (2010). The syncytium-specific expression of the Orysa;KRP3 CDK inhibitor: Implication of its involvement in the cell cycle control in the rice (*Oryza sativa* L.) syncytial endosperm. J. Exp. Bot..

[19] Yang R.F., Tang Q.C., Wang H.M., Zhang X.B., Pan G., Wang H., Tu J.M. (2011). Analyses of two rice (*Oryza sativa*) cyclin-dependent kinase inhibitors and effects of transgenic expression of OsiICK6 on plant growth and development. Ann. Bot. Lond..

[20] Zhou F., Lin Q., Zhu L., Ren Y., Zhou K., Shabek N., Wu F., Mao H., Dong W., Gan L. (2013). D14–SCFD3-dependent degradation of D53 regulates strigolactone signalling. Nature.

[21] Ma X., Zhang Q., Zhu Q., Liu W., Chen Y., Qiu R., Wang B., Yang Z., Li H., Lin Y. (2015). A Robust CRISPR/Cas9 System for Convenient, High-Efficiency Multiplex Genome Editing in Monocot and Dicot Plants. Mol. Plant.

[22] Bello B.K., Hou Y.X., Zhao J., Jiao G.A., Wu Y.W., Li Z.Y., Wang Y.F., Tong X.H., Wang W., Yuan W.Y. (2018). NF-YB1-YC12-bHLH144 complex directly activates Wx to regulate grain quality in rice (*Oryza sativa* L.). Plant Biotechnol. J..

[23] Fu F.F., Xue H.W. (2010). Coexpression analysis identifies Rice Starch Regulator1, a rice AP2/EREBP family transcription factor, as a novel rice starch biosynthesis regulator. Plant Physiol..

[24] Zhao L.F., Hu Y.B., Chong K., Wang T. (2010). ARAG1, an ABA-responsive DREB gene, plays a role in seed germination and drought tolerance of rice. Ann. Bot. Lond..

[25] Chastain C.J., Heck J.W., Colquhoun T.A., Voge D.G., Gu X.Y. (2006). Posttranslational regulation of pyruvate, orthophosphate dikinase in developing rice (*Oryza sativa*) seeds. Planta.

[26] Kang H.G., Park S., Matsuoka M., An G.H. (2005). White-core endosperm floury endosperm-4 in rice is generated by knockout mutations in the C-4-type pyruvate orthophosphate dikinase gene (OsPPDKB). Plant J..

[27] Kawakatsu T., Yamamoto M.P., Touno S.M., Yasuda H., Takaiwa F. (2009). Compensation and interaction between RISBZ1 and RPBF during grain filling in rice. Plant J..

[28] Deng Z.Y., Gong C.Y., Wang T. (2013). Use of proteomics to understand seed development in rice. Proteomics.

[29] Yu C.S., Chen Y.C., Lu C.H., Hwang J.K. (2006). Prediction of protein subcellular localization. Proteins.

[30] Schnittger A., Weinl C., Bouyer D., Schobinger U., Hulskamp M. (2003). Misexpression of the cyclin-dependent kinase inhibitor ICK1/KRP1 in single-celled *Arabidopsis* trichomes reduces endoreduplication and cell size and induces cell death. Plant Cell.

[31] Zhou Y., Wang H., Gilmer S., Whitwill S., Keller W., Fowke L.C. (2002). Control of petal and pollen development by the plant cyclin-dependent kinase inhibitor ICK1 in transgenic Brassica plants. Planta.

[32] Zhou Y., Fowke L.C., Wang H. (2002). Plant CDK inhibitors: Studies of interactions with cell cycle regulators in the yeast two-hybrid system and functional comparisons in transgenic *Arabidopsis* plants. Plant Cell Rep..

[33] Coelho C.M., Dante R.A., Sabelli P.A., Sun Y.J., Dilkes B.P., Gordon-Kamm W.J., Larkins B.A. (2005). Cyclin-dependent kinase inhibitors in maize endosperm and their potential role in endoreduplication. Plant Physiol..

[34] Pines J. (1995). Cyclins and cyclin-dependent kinases: A biochemical view. Biochem. J..

[35] Pines J. (1999). Four-dimensional control of the cell cycle. Nat. Cell Biol..

[36] Bird D.A., Buruiana M.M., Zhou Y., Fowke L.C., Wang H. (2007). *Arabidopsis* cyclin-dependent kinase inhibitors are nuclear-localized and show different localization patterns within the nucleoplasm. Plant Cell Rep..

[37] Zhou Y., Niu H., Brandizzi F., Fowke L.C., Wang H. (2006). Molecular control of nuclear and subnuclear targeting of the plant CDK inhibitor ICK1 and ICK1-mediated nuclear transport of CDKA. Plant Mol. Biol..

[38] Wang H., Qi Q., Schorr P., Cutler A.J., Crosby W.L., Fowke L.C. (1998). ICK1, a cyclin-dependent protein kinase inhibitor from *Arabidopsis thaliana* interacts with both Cdc2a and CycD3, and its expression is induced by abscisic acid. Plant J..

[39] Himanen K., Boucheron E., Vanneste S., de Almeida Engler J., Inze D., Beeckman T. (2002). Auxin-mediated cell cycle activation during early lateral root initiation. Plant Cell.

[40] Richard C., Lescot M., Inze D., De Veylder L. (2002). Effect of auxin, cytokinin, and sucrose on cell cycle gene expression in *Arabidopsis thaliana* cell suspension cultures. Plant Cell Tissue Organ Cult..

[41] Shu K., Liu X.D., Xie Q., He Z.H. (2016). Two Faces of One Seed: Hormonal Regulation of Dormancy and Germination. Mol. Plant.

[42] Xi W.Y., Liu C., Hou X.L., Yu H. (2010). MOTHER OF FT AND TFL1 Regulates Seed Germination through a Negative Feedback Loop Modulating ABA Signaling in *Arabidopsis*. Plant Cell.

[43] Xi W., Yu H. (2010). Mother Of ft and tfl1 regulates seed germination and fertility relevant to the brassinosteroid signaling pathway. Plant Signal Behav..

[44] Hu Y., Yu D. (2014). Brassinosteroid insensitive2 interacts with abscisic acid insensitive5 to mediate the antagonism of brassinosteroids to abscisic acid during seed germination in *Arabidopsis*. Plant Cell.

[45] Vaistij F.E., Gan Y.B., Penfield S., Gilday A.D., Dave A., He Z.S., Josse E.M., Choi G., Halliday K.J., Graham I.A. (2013). Differential control of seed primary dormancy in *Arabidopsis* ecotypes by the transcription factor SPATULA. Proc. Natl. Acad. Sci. USA.

[46] Finkelstein R.R., Gampala S.S., Rock C.D. (2002). Abscisic acid signaling in seeds and seedlings. Plant Cell.

[47] Qiu J.H., Hou Y.X., Tong X.H., Wang Y.F., Lin H.Y., Liu Q., Zhang W., Li Z.Y., Nallamilli B.R., Zhang J. (2016). Quantitative phosphoproteomic analysis of early seed development in rice (*Oryza sativa* L.). Plant Mol. Biol..

[48] Bleckmann A., Alter S., Dresselhaus T. (2014). The beginning of a seed: Regulatory mechanisms of double fertilization. Front. Plant Sci..

[49] Larkins B.A., Dilkes B.P., Dante R.A., Coelho C.M., Woo Y.M., Liu Y. (2001). Investigating the hows and whys of DNA endoreduplication. J. Exp. Bot..

[50] Van Leene J., Hollunder J., Eeckhout D., Persiau G., Van De Slijke E., Stals H., Van Isterdael G., Verkest A., Neirynck S., Buffel Y. (2010). Targeted interactomics reveals a complex core cell cycle machinery in *Arabidopsis thaliana*. Mol. Syst. Biol..

[51] Menges M., de Jager S.M., Gruissem W., Murray J.A. (2005). Global analysis of the core cell cycle regulators of *Arabidopsis* identifies novel genes, reveals multiple and highly specific profiles of expression and provides a coherent model for plant cell cycle control. Plant J..

[52] Mironov V.V., De Veylder L., Van Montagu M., Inze D. (1999). Cyclin-dependent kinases and cell division in plants—The nexus. Plant Cell.

[53] Shimotohno A., Matsubayashi S., Yamaguchi M., Uchimiya H., Umeda M. (2003). Differential phosphorylation activities of CDK-activating kinases in *Arabidopsis thaliana*. FEBS Lett..

[54] Hiei Y., Ohta S., Komari T., Kumashiro T. (1994). Efficient transformation of rice (*Oryza sativa* L.) mediated by Agrobacterium and sequence analysis of the boundaries of the T-DNA. Plant J..

[55] Hou Y., Wang L., Wang L., Liu L., Li L., Sun L., Rao Q., Zhang J., Huang S. (2015). JMJ704 positively regulates rice defense response against *Xanthomonas oryzae pv. oryzae* infection via reducing H3K4me2/3 associated with negative disease resistance regulators. BMC Plant Biol..

[56] Li B., Dewey C.N. (2011). RSEM: Accurate transcript quantification from RNA-Seq data with or without a reference genome. BMC Bioinform..

[57] Leng N., Dawson J.A., Thomson J.A., Ruotti V., Rissman A.I., Smits B.M.G., Haag J.D., Gould M.N., Stewart R.M., Kendziorski C. (2013). EBSeq: An empirical Bayes hierarchical model for inference in RNA-seq experiments. Bioinformatics.

[58] Livak K.J., Schmittgen T.D. (2001). Analysis of relative gene expression data using real-time quantitative PCR and the 2(-Delta Delta C(T)) Method. Methods.

[59] Lin Q.B., Wu F.Q., Sheng P.K., Zhang Z., Zhang X., Guo X.P., Wang J.L., Cheng Z.J., Wang J., Wang H.Y. (2015). The SnRK2-APC/C-TE regulatory module mediates the antagonistic action of gibberellic acid and abscisic acid pathways. Nat. Commun..

[60] Wang X.F., Wang Y.F., Pineros M.A., Wang Z.Y., Wang W.X., Li C.Y., Wu Z.C., Kochian L.V., Wu P. (2014). Phosphate transporters OsPHT1;9 and OsPHT1;10 are involved in phosphate uptake in rice. Plant Cell Environ..

