# Supplementary figures and images for: Cyclin-Dependent Kinase Inhibitors KRP1 and KRP2 Are Involved in Grain Filling and Seed Germination in Rice (Oryza sativa L.)
Source: Int J Mol Sci. 2019 Dec 30;21(1):245. doi: 10.3390/ijms21010245 (PMC6981537; doi:10.3390/ijms21010245)

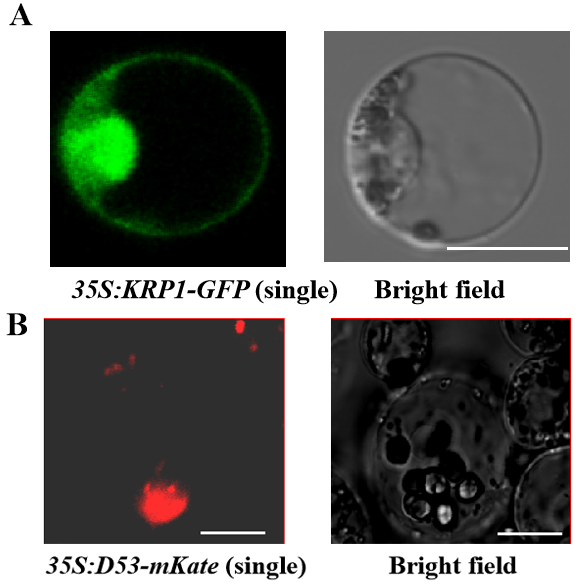

Supplement: Supplementary file 1 [file ijms-21-00245-s001.zip › Figure S1.tif]

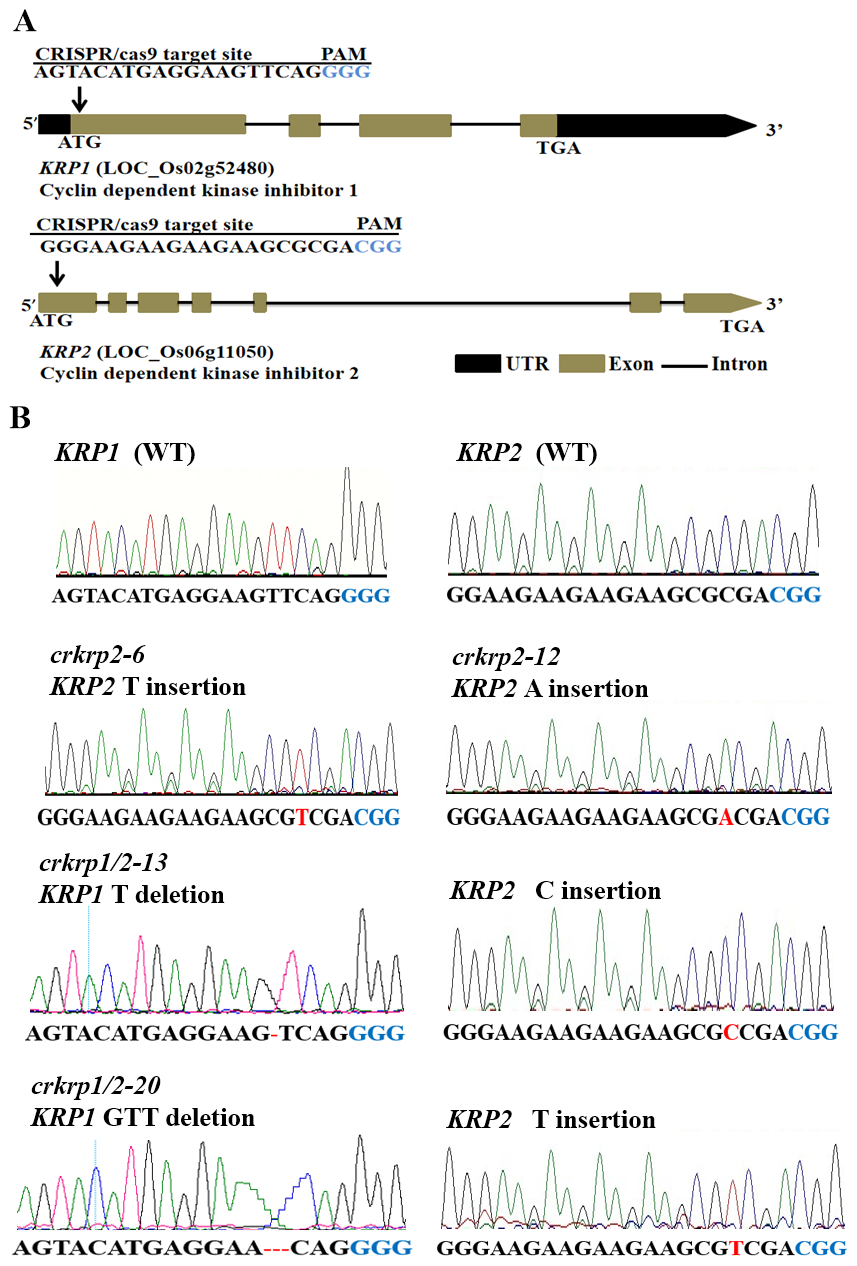

Supplement: Supplementary file 1 [file ijms-21-00245-s001.zip › Figure S2.tif]

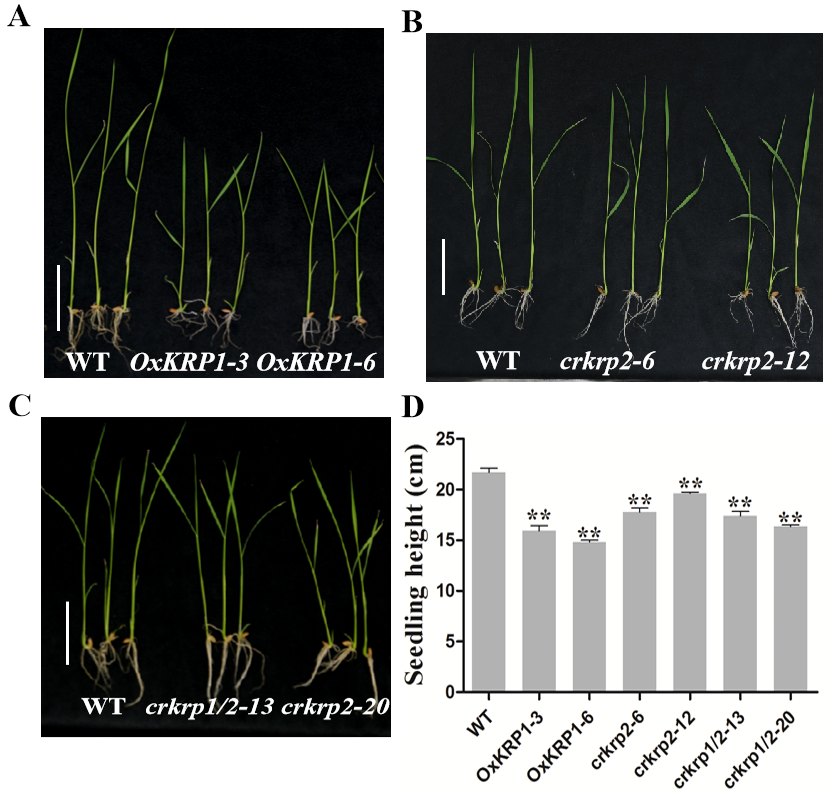

Supplement: Supplementary file 1 [file ijms-21-00245-s001.zip › Figure S3.tif]

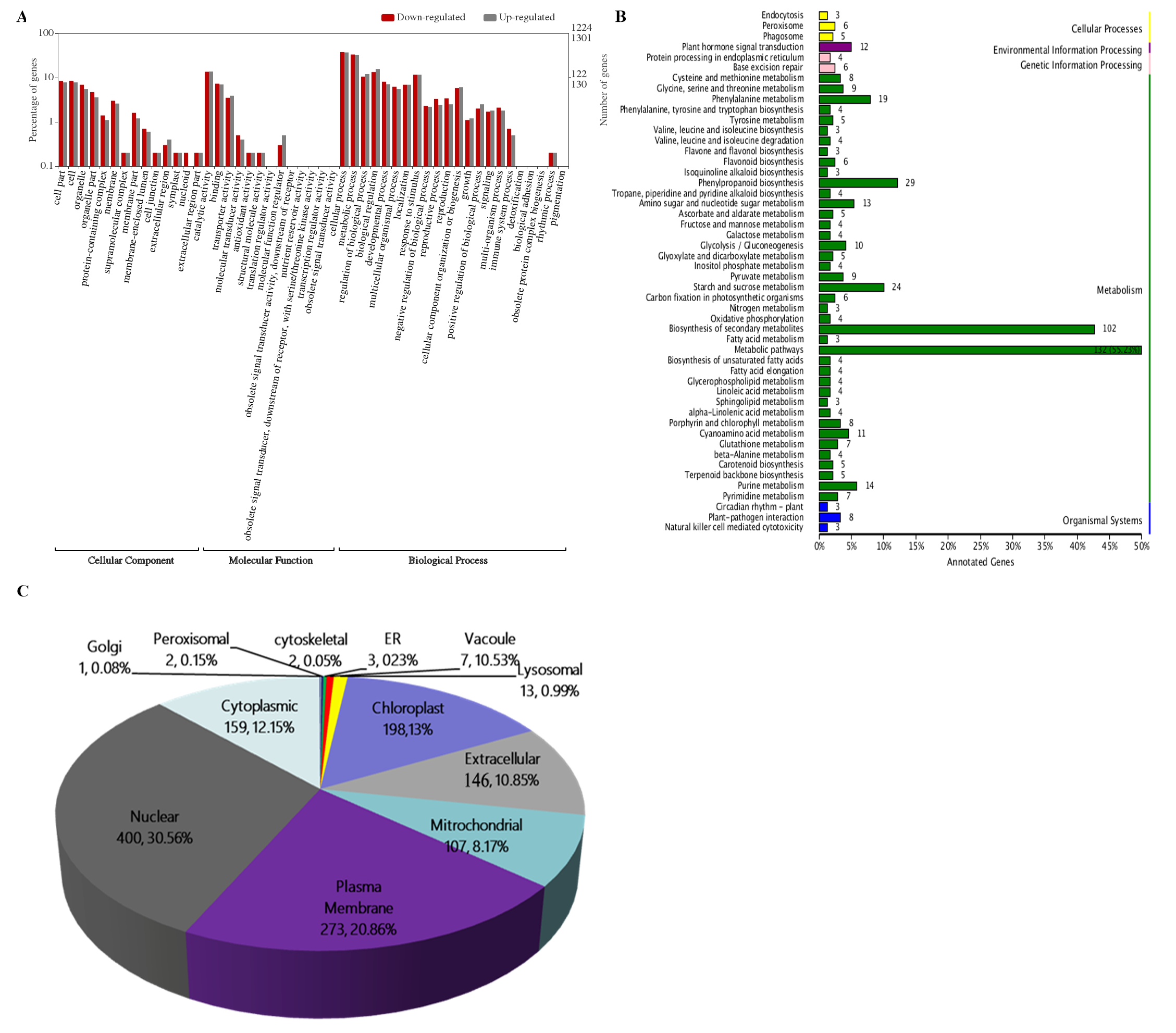

Supplement: Supplementary file 1 [file ijms-21-00245-s001.zip › Figure S4.tif]
